# Prolonged exposure to bacterial toxins downregulated expression of toll-like receptors in mesenchymal stromal cell-derived osteoprogenitors

**DOI:** 10.1186/1471-2121-9-52

**Published:** 2008-09-18

**Authors:** Irene Fung Ying Mo, Kevin Hak Kong Yip, Wing Keung Chan, Helen Ka Wai Law, Yu Lung Lau, Godfrey Chi Fung Chan

**Affiliations:** 1 Department of Paediatrics and Adolescent Medicine, Faculty of Medicine, The University of Hong Kong, Hong Kong, PR China; 2 Discipline of Prosthodontics, Faculty of Dentistry, The University of Hong Kong, Hong Kong, PR China; 3 Associate Professor & Honorary Consultant & Assistant Dean (External Affair), Department of Paediatrics and Adolescent Medicine, Queen Mary Hospital, 102 Pokfulam Road, The University of Hong Kong, HKSAR, PR China

## Abstract

**Background:**

Human mesenchymal stromal cells (MSCs, also known as mesenchymal stem cells) are multipotent cells with potential therapeutic value. Owing to their osteogenic capability, MSCs may be clinically applied for facilitating osseointegration in dental implants or orthopedic repair of bony defect. However, whether wound infection or oral microflora may interfere with the growth and osteogenic differentiation of human MSCs remains unknown. This study investigated whether proliferation and osteogenic differentiation of MSCs would be affected by potent gram-positive and gram-negative derived bacterial toxins commonly found in human settings.

**Results:**

We selected lipopolysaccharide (LPS) from *Escherichia coli *and lipoteichoic acid (LTA) from *Streptococcus pyogenes *as our toxins of choice. Our findings showed both LPS and LTA did not affect MSC proliferation, but prolonged LPS challenge upregulated the osteogenic differentiation of MSCs, as assessed by alkaline phosphatase activity and calcium deposition. Because toll-like receptors (TLRs), in particularly TLR4 and TLR2, are important for the cellular responsiveness to LPS and LTA respectively, we evaluated their expression profiles serially from MSCs to osteoblasts by quantitative PCR. We found that during osteogenic differentiation, MSC-derived osteoprogenitors gradually expressed TLR2 and TLR4 by Day 12. But under prolonged incubation with LPS, MSC-derived osteoprogenitors had reduced TLR2 and TLR4 gene expression. This peculiar response to LPS suggests a possible adaptive mechanism when MSCs are subjected to continuous exposure with bacteria.

**Conclusion:**

In conclusion, our findings support the potential of using human MSCs as a biological graft, even under a bacterial toxin-rich environment.

## Background

Mesenchymal stromal cells (MSCs) have been successfully extracted from bone marrow, muscle, adipose tissue, placenta, and umbilical cord blood [1]. MSCs have also been found in both human adult dental pulp and exfoliated deciduous teeth [2-4]. Bone marrow-derived MSCs are characterized by a high proliferative capacity *ex vivo *and can differentiate into a wide variety of mesenchymal progenitor cells under defined culture conditions [5-8]. The potential therapeutic value of MSCs in regenerative medicine, tissue engineering, and cellular therapy has attracted substantial interest in recent years [1].

Because MSCs can be induced to differentiate into osteogenic tissues [9], their application to modern dentistry and orthopedic surgery as an alternative to autologous bone grafts has been explored [2,10]. Accordingly, most studies focused on using MSCs for *in vitro *or *in vivo *tissue regeneration in the setting of osseointegration of dental implants or bone grafts for either alveolar bone or long bone reconstruction [10-13]. However, the human oral cavity contains a high density of microflora, and dental implants grafted with MSCs are hence continuously exposed to different bacterial species and their toxins. In addition, MSC-aided dental implants or bone grafts might encounter periodontopathic bacteria-causative agents of periodontitis. The feasibility of using MSCs in osseointegration by virtue of their differentiation into osteoblasts depends on whether the growth and osteogenic differentiation of MSCs is affected by bacterial stimuli. Yet, little is known about the interactions between bacteria and MSCs. Most studies on this subject focused on the effects of periodontopathic bacteria on osteoclasts, a key cell population involved in bacteria-induced bone destruction. Only a few studies have been done on osteoblasts [14-16] or its progenitors [17-19], or MSCs [20-22]. Therefore, this study aimed to investigate the effect of purified bacterial toxins on the proliferation and osteogenic differentiation of MSCs.

*Streptococcus pyogenes *is a gram-positive bacterium that is commonly found in the human oral cavity; and *Escherichia coli *is a gram-negative bacterium that is commonly associated with clinical sepsis or occassionally osteomyelitis [14]. Lipopolysaccharide (LPS) and lipoteichoic acid (LTA) are bacterial cell-wall components that are derived from these gram-negative and gram-positive bacteria, respectively. LPS triggers immune responses such as proliferation and release of proinflammatory cytokines from human immune cells. LTA shares with LPS many of its pathophysiological properties and can thus be considered as the gram-positive counterpart of LPS but with a lower mitogenicity [23]. The signaling pathways of these two bacterial cell-wall components are initiated through the Toll-like receptors (TLRs) on the surface of immune cells such as macrophages and dendritic cells. TLRs are specific receptors that respond to specific pathogen-associated molecular patterns on microbial pathogens by activating innate immune responses and triggering host defense mechanisms [24,25]. There are at least 11 different TLRs found in mammals [25,26]; among them, TLR4 is the major LPS receptor [27], and TLR2 (along with TLR1/TLR6) is predominantly responsible for recognizing gram-positive cell-wall structures such as LTA [28]. Although the expression of TLRs has been thoroughly investigated in immune cells, only several studies in recent 3 years studied about the gene or protein expression of TLRs in human bone marrow-derived MSCs [20,29-32]. Previous studies demonstrated that osteoblastic cells or MSCs express TLR2, TLR4, and other molecules involved in LPS signaling pathways (Table 1).

**Table 1:** Expression of genes encoding LPS receptor and signaling molecules in osteoblastic cells

Cell type	TLR2	TLR4	MD2	CD14	MyD88	References
SaOS-2, HOS, MG-63	-	+^*a*^	+	+	+	[46]
MC3T3-E1	+	+	NA	NA	NA	[40]
ST-2	+	+	NA	NA	NA	[40]
SaM-1 cell	NA	NA	NA	+	NA	[47]
Primary mouse	+	+	NA	+	NA	[48]
osteoblasts from	+	+	NA	NA	NA	[40]
embryonic calvaria	NA	NA	NA	+	NA	[49]

+, constitutively expressed; -, not detected; NA, not available.

SaOS-2, HOS, MG-63: human osteosarcoma cell-lines; MC3T3-E1: mouse osteoblastic cell-line; ST-2: mouse stromal cell-line; SaM-1: human osteoblastic cell-line from periosteum

TLR2: Toll-like receptor 2; TLR4: Toll-like receptor 4.

^*a *^Protein expression of TLR4 was also observed for SaOS-2 only.

In this study, we examined the effects of LPS and LTA on the proliferation and osteogenic differentiation of MSCs. Then, we explored TLR2 and TLR4 gene expression profiles during the osteogenic differentiation of MSCs with and without the presence of relevant bacterial toxins.

## Results

### LPS and LTA did not induce MSC proliferation

**Figure 1:**
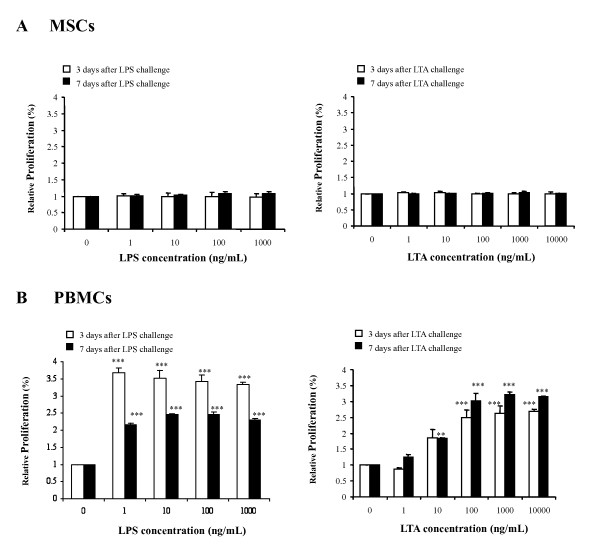
**Effects of LPS and LTA on the proliferation of human MSCs and PBMCs**. Cultured cells were incubated with either LPS (left panel) or LTA (right panel) for 3 days or 7 days. On Day 3 and Day 7, the cell proliferation responses of (**A**) MSCs and (**B**) PBMCs were assessed by XTT assay respectively. LPS and LTA did not affect the proliferation of MSCs (pooled data from three experiments, each done in triplicates) but increased proliferation of PBMCs (one representative experiment done in triplicates). The data are expressed as the relative proliferation of untreated controls, mean ± SEM. * *P *< 0.05; ** *P *< 0.01; *** *P *< 0.001 versus control.

The multipotency of MSCs used in this investigation was confirmed by standard differentiation assays along three lineages including osteogenic, adipogenic and chondrogenic. All the MSCs from the three donors showed similar differentiating functions. We then tested whether the MSCs responded to bacterial toxins LPS or LTA. As shown in Figure 1A, LPS (left panel) and LTA (right panel) did not affect the proliferation of MSCs during either the 3- or 7-day period, even after incubation with a relatively high dose of toxin (1 μg/mL of LPS and 10 μg/mL of LTA). As expected, these toxins evoked a marked increase in proliferation of peripheral mononuclear cells (PBMCs) in compared to untreated controls (Figure 1B) (n = 3, *P *< 0.01). For the response of PBMCs to bacterial toxins, 1 ng/mL LPS was sufficient to induce a significant increase in proliferation on Day 3 and Day 7. Since LTA is less potent as compared to LPS, higher dosages of LTA, 10 ng/mL and 100 ng/mL, were needed to generate a significant response on Day 7 and Day 3 respectively.

### Prolonged LPS challenge enhanced osteogenic differentiation of MSCs

**Figure 2:**
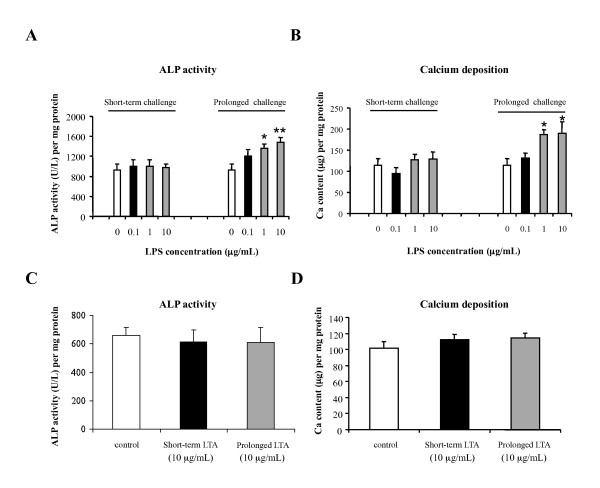
**Effects of LPS and LTA on the osteogenic differentiation of MSCs**. MSCs were cultured in osteogenesis-induction medium and exposed to toxin for 3 days or for the whole incubation period. According to ALP activity and calcium deposition assays performed on Day 10 (LPS) or 14 (LTA), short-term LPS challenge did not affect the osteogenic differentiation (**A **and **B**), but prolonged challenge to 1 and 10 μg/mL upregulated ALP activity and calcium deposition. Neither short-term nor prolonged LTA challenge affected the osteogenic differentiation (**C **and **D**). The data are from three independent experiments and expressed as mean ± SEM. *P < 0.05; ** *P *< 0.01 versus control.

To study the effect of bacterial toxins on the osteogenic differentiation of MSCs, we exposed MSCs with osteogenesis-induction medium and toxin for a short-term of 3 days, or prolonged period of either 10 or 14 days as time points for respective quantitative assays. Alkaline phosphatase (ALP) activity was used as an early marker of osteogenic differentiation [33,34]. A period of 3 days was chosen as the duration for the short-term challenge because a significant difference in ALP activity between MSCs with and without osteogenic induction has been reported previously on Day 4 after osteogenic induction [9]. Prolonged toxin challenge lasted 10 to 14 days to allow optimal quantification of MSC osteogenic differentiation by ALP and calcium assays respectively based on our preliminary studies. Under short-term toxin challenge, neither LPS at concentrations of 0.1, 1, and 10 μg/mL (Figure 2A and 2B) nor LTA at 10 μg/mL (Figure 2C and 2D) had a significant effect on ALP activity or calcium deposition. After prolonged LPS challenge of higher doses (1 and 10 μg/mL), ALP activity and calcium levels were significantly higher than those in untreated controls (n = 3, *P *< 0.05) (Figure 2A and 2B). In contrast, prolonged LTA challenge had no effect on ALP activity or calcium level (Fig 2C and 2D). The increased calcium deposition after prolonged LPS challenge was further confirmed semi-quantitatively by von Kossa staining (data not shown).

### Basal levels of TLR4 and TLR2 expression in MSCs were low

**Figure 3:**
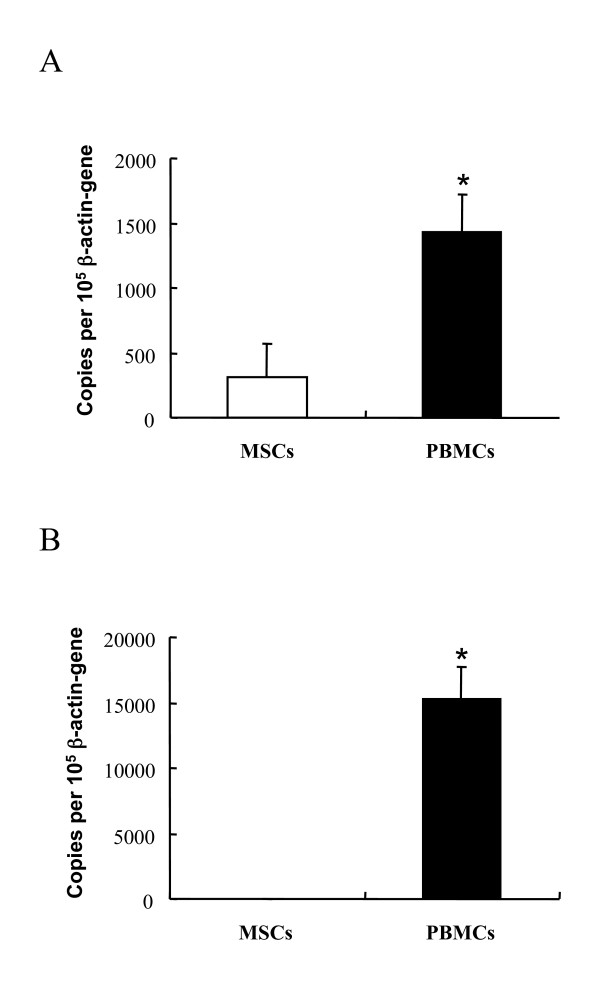
**TLR4 and TLR2 constitutive gene expression in MSCs and PBMCs**. Quantitative PCR revealed that constitutive expression of (**A**) TLR4 was higher in PBMCs than in MSCs, while (**B**) TLR2 was not expressed in MSCs. The data are pooled from three experiments, each done in duplicates, and expressed as mean ± SEM. * *P *< 0.05; ** *P *< 0.01.

Because only the osteogenic function but not the proliferation of MSCs was affected by LPS, we hypothesized that MSC may not express TLR4 (the major LPS receptor) until osteogenic differentiation is triggered. We thus evaluated TLR4 and TLR2 mRNA levels by quantitative PCR. The constitutive gene expressions of TLR4 and TLR2 on MSCs were determined using PBMCs as a positive control because these cells express all TLRs constitutively. As expected, the basal level of TLR4 mRNA in MSCs was much lower than that of PBMCs (n = 3, *P *< 0.05) (Figure 3A). A similar result was observed for the TLR2 mRNA level, which was virtually undetectable in MSCs (n = 3, *P *< 0.01) (Figure 3B).

### MSC-derived osteoprogenitors expressed TLR4 and TLR2 during osteogenesis

**Figure 4:**
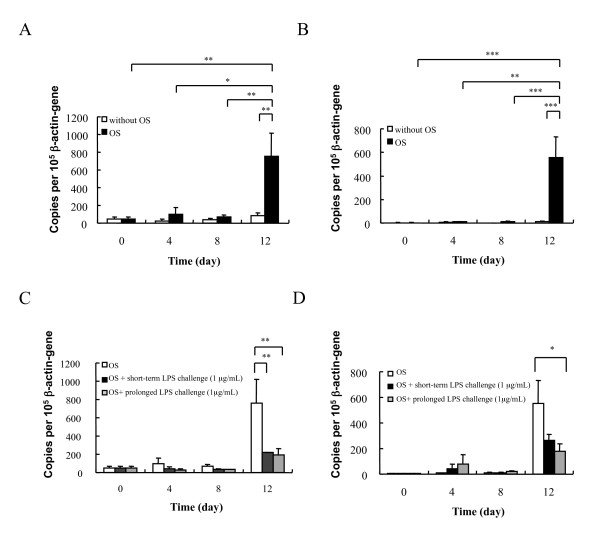
**TLR gene expression of MSCs at basal state and under osteogenic differentiation with and without LPS exposure**. (**A**) Quantitative PCR revealed that TLR4 expression was significantly higher after 12 days of incubation in osteogenesis-induction medium than after shorter periods, and higher than after 12 days of constitutive expression. (**B**) Quantitative PCR revealed that TLR2 expression in MSCs was significantly higher after 12 days of incubation in osteogenesis-induction medium than after shorter periods, and higher than after 12 days of constitutive expression. (**C**) TLR4 expression in MSCs cultured under osteogenic conditions was lower on Day 12 after exposure to LPS (1 μg/mL) for 3 or 12 days. (**D**) TLR2 expression in MSCs cultured under osteogenic conditions was lower on Day 12 after exposure to LPS (1 μg/mL) for 12 days, but not after 3-day LPS exposure. The data are pooled from three experiments, each done in duplicates, and expressed as mean ± SEM. * *P *< 0.05; ** *P *< 0.01; *** *P *< 0.001.

When we monitored the expression of TLR4 and TLR2 during MSC osteogenic differentiation, TLR4 mRNA levels were low during the initial 8 days but increased sharply on Day 12, by about 9-fold compared to control (n = 3, *P *< 0.01) (Figure 4A). A similar expression pattern was observed for TLR2 mRNA levels, with an increase of about 48-fold. (n = 3, *P *< 0.001) (Figure 4B). Without osteogenic induction, TLR2 and TLR4 expression were very low.

### LPS challenge downregulated TLR4 and TLR2 expression in MSC-derived osteoprogenitors

To determine the effect of LPS on TLR4 expression during MSC osteogenic differentiation, we exposed MSCs to LPS (1 μg/mL) under osteogenic conditions. LPS was chosen for we did not find any effect induced by LTA based on our result mentioned. The TLR4 and TLR2 mRNA levels were then determined by quantitative PCR on Day 0, 4, 8, and 12 after osteogenic induction. Under both short-term (3-day) and prolonged LPS challenge, TLR4 expression of MSC-derived osteoprogenitors was downregulated significantly, by about 75%, on Day 12 (n = 3, *P *< 0.01) (Figure 4C). Surprisingly, even the level of TLR2 expression was also suppressed by prolonged LPS challenge on Day 12, by about 67% (n = 3, *P *< 0.05) (Figure 4D).

## Discussion

The increasing interest in using MSCs as a source of cellular therapy means that the potential impact of bacterial toxins on the growth and differentiation of MSCs will be a growing concern. Exposure to endotoxin-enriched environment may affect many aspects of MSCs properties such as self-renewal, differentiation potential, production of cytokines & ECM compound. Our study focused on the investigation of the effects of two purified bacteria-derived toxins on the growth and osteogenic differentiation of human MSCs. Cell proliferation was not affected by LPS or LTA, and osteogenic differentiation was promoted by prolonged exposure to LPS but not to LTA. In addition, brief exposure to LPS during osteogenic differentiation reduced the expression of TLR4, whereas prolonged exposure reduced the expression of both TLR4 and TLR2.

Most previous related studies have been conducted on osteoclasts [35-38], than osteoblasts [14-16] or their committed progenitors [17-19]. Among the studies related to the bacterial effects on the osteogenic progenitors, they used either a 6-day chick periosteal osteogenesis model or rat calvaria cells, which contain a subpopulation of osteogenic precursors. The objectives of these studies were to find out the possible adverse effects of bacterial toxin on osteogenesis and they did show bacterial toxin impaired the osteogensis process. We studied the effect of bacterial toxins on osteogenic differentiation using human MSCs from bone marrow aspirate, which is an enriched source of multipotential stem cells. In addition, it is a novel model comparing to the chick or rat models for it allowed us to study how bacterial toxins might affect osteogenic differentiation from MSCs to osteogenic progenitors.

Our study showed that neither LPS nor LTA affected the proliferation of MSCs, even at relative high endotoxin doses. This is in line with the findings of previous study by Hwa Cho et al [29]. We then explored whether these responses pattern may be related to the expression levels of TLR4 and TLR2 genes in MSCs. This hypothesis was partly supported by our observation that very low level of TLR4 nor TLR2 mRNAs expression levels were detectable in MSCs. These low TLR4 and TLR2 expression profiles may serve as a protective mechanism to maintain stem cell survival from the effect of bacterial toxins, even at high concentrations. For cellular therapy purpose, this suggested that MSCs could be safely used in clinical settings even in the microenvironment with bacteria such as oral surgical sites.

TLR4 and TLR2 expressions in MSC-derived osteoprogenitors were minimal during early osteogenic induction but emerged by Day 12. The expression of these two genes seems to be a natural part of osteoblast differentiation in the absence of external bacterial stimuli. This finding agrees with those of previous studies in which osteoblastic cells and osteoblasts constitutively express TLR4, TLR2, and other LPS-signaling molecules (Table 1). This is slightly different from what was observed previously which showed TLR-2, 3, 4, 6 had a higher expression as compared to TLR-1, 5, 9, but such differences may be related to different type of methodology being adopted [29]. Another study on murine BM-derived MSCs also showed TLR1 to TLR8 but not TLR9 mRNA was expressed [30]. Whether such variations in TLRs expression profile has something to do with the state of differentiation require further investigation. Although previous studies examined the basal TLR expression level in MSCs and how they affect MSC differentiation as we did, our study provided information on the real time dynamic changes in the TLR2 and TLR4 mRNA expression profile as they underwent osteogenic differentiation from MSCs under the influence of LPS or LTA. Interestingly, LPS but not LTA could also downregulate the TLR2 and 4 expressions on Day 12 of differentiation, no matter it was under either short or prolonged LPS exposure. Our findings suggested that the TLR2 and 4 expressions could be negatively regulated by endotoxin and such phenomenon has been described in some immune cells such as monocytes.

When testing whether LPS and LTA affect the osteogenic differentiation of human MSCs, we found that short-term challenge by either toxin had no effect, but prolonged LPS challenge upregulated both ALP activity and calcium deposition. This is in line with the findings of Hwa Cho et al. who found an increase in the osteogenic marker genes expression such as the ALP, osteopontin and BMP2 at the 5th and 10th day after induction [29]. We confirmed such findings by measuring the ALP protein level on Day 10.

Documented effects of *E. coli *LPS on osteoblastic cells are mixed. No inhibitory effect of *E. coli *LPS were found on osteoblastic cell line MC3T3-E1 and rat calvaria cells [17,39]. However, Shjoi et al. found an inhibitory effect on ALP activity of SaOS-2, an osteoblast-like osteosarcoma cell line [15]. Factors contributing to discrepancies in results may include heterogeneity of the cell source, the experimental conditions, and the culture system used. For TLR2 ligands, it has been shown that PGN stimulated the osteogenic differentiation of human adipose-derived MSCs in a dose-dependent manner similar to that of LPS [29]. However, no such effect was found concerning LTA used in our study on BM-derived MSCs. Why LPS and LTA affect the MSC differentiation differently in our study remains to be answered, but the specificity and potency of different ligands on the TLRs and also the involvement of different signaling pathways may account for such variations [20,30-32].

In this study, the effects of LPS exposure during MSC osteogenic differentiation depended on the duration of exposure. This finding might be explained partly by the kinetics of TLR4 gene expression: the lack of an effect from short-term LPS challenge might be due to the delay in expression of TLR4. Therefore, the timing of the activation of TLR genes may play an essential role in altering the osteogenic activity of MSC-derived osteoprogenitors. This finding may explain why studies using osteoblasts and osteogenic precursor cells as effector cells can yield different results after bacterial challenge.

On the other hand, since osteogenic differentiation of MSCs was promoted by prolonged LPS challenge, we further examined whether constitutive TLR gene expression on osteoprogenitors was also affected by LPS. Interestingly, we found that expression of both TLR4 and TLR2 genes was downregulated in osteoprogenitors after continuous LPS exposure. Little has been reported on how bacterial toxins regulate the expression of TLRs on osteoblastic cells. One study [40] showed an immediate effect of LPS challenge on TLR2 and TLR4 expression of primary murine osteoblasts and MC3T3-E1 cells; unexpectedly, TLR2 but not TLR4 mRNA was upregulated within two hours of *E. coli *LPS exposure. This raised a question about the specificity of TLRs and some degree of crosstalk between TLR2 and TLR4 may actually exist. In our experiments, prolonged toxin exposure was designed to mimic the situation in which MSCs interact continuously with oral microflora or with bacterial toxins during chronic inflammatory bone disease. Our results suggested that MSC-derived osteoprogenitors could adapt to continuous LPS challenge by reducing TLR4 and TLR2 expression, thereby they can be spared from the toxic effect of these toxins, leading to a paradoxical upregulation of osteogenic activities.

A similar adaptive response phenomenon, known as "endotoxin tolerance," can be found in monocytes and macrophages [41]. Endotoxin tolerance has been well documented as a cell-desensitizing phenomenon that results from sustained exposure of sublethal doses of LPS, which leads to a reduced capacity of the host (*in vivo*) or of macrophages (*in vitro*) to further respond to LPS [42]. Furthermore, TLR4 expression on the surface of LPS-tolerant macrophages has been shown to be downregulated [42], which may account for the molecular mechanism of endotoxin tolerance. Therefore, we suggest that MSC-derived osteoprogenitors acquire an adaptive tolerance under continuous LPS exposure in order to prevent excessive bone and tissue destruction, thereby protecting or preserving the host organ tissues.

Endotoxin tolerance can be found in oral mucosa cells and is associated with chronic periodontitis [43]. In addition, TLR2 and TLR4 mRNAs are significantly downregulated in the gingival tissue of patients with chronic periodontitis as compared to healthy persons [43], suggesting that the oral mucosa develops endotoxin tolerance in chronic periodontitis. Although experimental endotoxin tolerance involves prior incubation of LPS before subsequent challenge, a study of human gingival fibroblasts-a major constituent of gingival connective tissue that interacts directly with bacteria in periodontitis-has shown that TLR4 expression can be downregulated by LPS without prior LPS incubation [44]. Whether osteoprogenitors or osteoblasts indeed display endotoxin tolerance remains to be confirmed. Interestingly, we also observed downregulation of TLR4 gene expression after osteogenic induction in MSCs that had undergone short-term LPS challenge. These data may suggest that gene might be switched off in the early phase of osteogenic differentiation. Finally, the paradoxical puzzle of inhibition of the TLR receptor expression by LPS was not contradictory to the positive effect on osteogenic differentiation remained to be solved. Possible explanation may include the presence of alternated receptor and signaling pathway for LPS induced osteogenesis.

## Conclusion

In conclusion, we found that MSCs initially express very low levels of TLR2 and TLR4, but MSC-derived osteoprogenitors acquire these receptors. Such dynamic changes may at least partly explain why osteoblasts become responsive to LPS after an early critical stage of osteogenic differentiation. More importantly, MSC-derived osteoprogenitors adapt by reducing TLR4 and TLR2 expression after exposure to LPS during osteogenic differentiation. This adaptive response suggests that an intrinsic regulatory mechanism maintains homeostasis in tissues that persistently interact with microbial flora such as in the oral mucosa or infected tissue. These findings support the potential of using human MSCs as a biological graft, even in a bacterial toxin-rich environment. However, it is still not clear from our data that whether other properties of MSCs such as cytokine release, cell adhesion, etc. are affected by exposure to endotoxins and further works are needed to clarify on these aspects.

## Methods

### Isolation and culture of human MSCs

This study was approved by the Joint Ethics Committee (Internal Review Board) of The University of Hong Kong and the Hospital Authority Hong Kong West Clusters. The culture conditions, immunophenotyping, and assays for confirming the differentiating functions of human MSCs have been described previously [45]. Briefly, bone marrow samples were collected from three young healthy adult bone marrow donors. All MSCs were only used within 3–6 passages. Heparinized bone marrow samples were mixed with twice their volume of phosphate-buffered saline (PBS) and were separated in Ficoll-Hypaque density gradient (Amersham Biosciences, Uppsala, Sweden). Mononuclear cells were collected from the interface and washed twice with PBS. The washed cells were resuspended in MSC medium, which consisted of low-glucose Dulbecco's modified Eagle medium (DMEM; Invitrogen, Carlsbad, CA), 10% fetal bovine serum (FBS; Hyclone, Logan, UT), 100 U/mL penicillin, 100 mg/mL streptomycin, and 2 mM L-glutamine (Invitrogen). The cells were plated at 5 to 30 × 10^6^cells in 100-mm^2 ^culture dishes. Cultures were maintained at 37°C in a humidified atmosphere containing 5% CO_2_. After 24 h, nonadherent cells were removed and the adherent cells were washed twice with PBS. The culture medium was replaced every three to four days. When the cultures approached confluence, cells were detached with a solution of 0.05% trypsin and 25 mM EDTA (Invitrogen) and replated at a density of 2 × 10^5 ^cells in 75-cm^2 ^culture flasks. By using flow cytometry, all the MSCs used in the studies have standard MSCs immunophenotypes with positive for CD29, CD105, CD73, CD90 and MHC class I; negative for CD45, CD34, CD14, CD19 and MHC class II.

### Isolation and culture of human peripheral blood mononuclear cells

PBMCs were used as a positive control and were isolated from the buffy coat derived from blood taken from healthy voluntary donors. The buffy coat was diluted with PBS and separated in a Ficoll-Hypaque gradient density. Mononuclear cells were collected from the interface and washed twice with PBS. The washed cells were resuspended in RMPI 1640 medium that was supplemented with 10% FBS, 100 U/mL penicillin, and 100 mg/mL streptomycin. Cultures were maintained at 37°C in a humidified atmosphere containing 5% CO_2_.

### Cell proliferation assay

The effects of LPS and LTA on MSCs and PBMCs proliferation were measured using Cell Proliferation Kit II XTT assay kit (Roche Molecular Biochemicals, Mannheim, Marburg, Germany), which was based on the mitochondrial activity of metabolic active cells, according to the manufacturer's instructions. The findings were further correlated with cell counting. Briefly, cells were grown in flat-bottomed 96-well plates in a final volume of 100 μL of culture medium per well. LPS from *E. coli *O111:B4 or LTA from *S. pyogenes *(Sigma, St. Louis, MO) were then added to the culture medium at different concentrations (LPS, 1 ng/mL-1 μg/mL; LTA, 1 ng/mL-10 μg/mL) for 3 or 7 days for XTT assay. After the incubation period, 50 μL of the XTT labeling mixture was added to each well and incubated for 4 h at 37°C in a humidified atmosphere containing 5% CO_2_. The spectrophotometric absorbance was measured at 450 nm, and results were normalized against that of untreated MSCs and expressed as the relative proliferation (%).

### Differentiation of MSCs

The capacity of MSCs to differentiate along osteogenic and adipogenic lineages was assessed as previously reported [5-8]. For the induction of MSC osteogenic differentiation, the cells were treated with osteogenesis-induction (OS) medium, which was DMEM containing 10% FBS, 50 μM L-ascorbic acid-2-phosphate, 10 mM β-glycerol phosphate, and 100 nM dexamethasone (Sigma, St. Louis, MO). Differentiation was monitored with alkaline phosphatase (ALP) and calcium spectrophotometric assays; ALP activity and calcium deposition were also tested by ALP staining and von Kossa staining.

For the induction of MSC adipogenic differentiation, confluent MSC cultures were induced to undergo adipogenic differentiation by culturing cells in adipogenesis-induction (MDI+I) medium, which was DMEM containing 1 μM dexamethasone, 10% FBS, 0.5 mM methyl-isobutylxanthine, 10 μg/mL insulin, and 100 μM indomethacin, 100 U/mL penicillin, and 100 mg/mL streptomycin (Sigma). After 48 to 72 h, the medium was changed for 24 h to adipogenesis-maintenance (AM) medium, which was DMEM containing insulin (10 μg/mL), 10% FBS, 100 U/mL penicillin, and 100 mg/mL streptomycin (Sigma). The MSCs were then retreated with MDI+I and AM medium alternately for two more cycles. The cells were stained with Oil Red O to reveal the extent of lipid accumulation.

For the induction of MSC chondrogenic differentiation, aliquots of 2 × 10^5 ^cells in a 15 mL centrifuge tube were centrifuged at 800 rpm for 5 min at room temperature. The pellet was resuspended in chondrogenesis-induction medium, which was DMEM high glucose containing 10 ng/mL recombinant human transforming growth factor beta 3 (rhTGF-β3), 100 nM dexamethasone, 6 mg/mL insulin, 100 mM ascorbic acid 2-phosphate, 1 mM sodium pyruvate, 6 mg/mL transferrin, 0.35 mM praline and 1.25 mg/mL bovine serum albumin (Sigma). Cells were then centrifuged as pellet and maintained for 3 weeks with regular induction medium replacement every 2 days. At the end of the incubation, pellets were fixed and processed for 5 μM thick paraffin sections for Alcian blue staining.

### Exposure of MSCs to bacterial toxins under osteogenic conditions

MSCs were plated in MSC medium at 2 × 10^3 ^cells/cm^2 ^in 6 well-plates. On the following day (Day 0), the cells were incubated with osteogenesis-induction (OS) medium and toxins. The concentrations of LPS (0.1, 1, and 10 μg/mL) and LTA (10 μg/mL) used were based on preliminary data. Incubation with bacterial toxin was either short-term (3-day) or continuous until specific endpoints for quantification of osteogenic differentiation. After the short-term exposure to bacterial toxins, the cells were washed and replaced with culture medium. All cells (either for short-term or prolonged exposure to toxins) will be subjected to the determination of alkaline phosphatase (ALP) activity on Day 10 and calcium deposition on Day 14. The medium and toxin were replaced every 3 day.

### ALP assay

On Day 10 after osteogenic induction in the presence or absence of toxins, an ALP assay was performed colorimetrically using a Liquicolor kit (Stanbio Laboratory, Boerne, TX). To prepare the cell lysates, cell layers were washed twice with PBS and then extracted with 1% Triton X-100 (v/v, in water) by shaking overnight at 4°C. ALP enzyme activity was calculated after spectrophotometrically measuring the absorbance at 405 nm of p-nitrophenol product that formed at 37°C. ALP activity was defined as the amount of enzyme that produced 1 mM of p-nitrophenol per minute. Results were recorded as ALP activity per milligram of total cell protein.

### Calcium assay

On Day 14 after osteogenic induction in the presence or absence of toxins, cell layers were rinsed twice with PBS and scraped off in 0.5 M HCl. The cell layers were extracted by shaking overnight at 4°C and then centrifuged at 1000 × g for 5 min. The supernatant was used for calcium level determination according to the manufacturer's instructions using a Liquicolor kit (Stanbio Laboratory). Calcium deposition was determined by absorbance measured spectrophotometrically at 630 nm; standards were prepared in parallel. Results were expressed as amount of calcium (μg) per milligram of total cell protein.

### Determination of TLR4 and TLR2 mRNA levels

To study TLR4 and TLR2 gene expression profiles of MSCs with and without osteogenic induction, we collected RNA on Day 0, 4, 8, and 12 with and without OS medium. MSCs under osteogenic conditions were also exposed to either short-term (3-day) or continuous challenge to toxin until sample collection. After the initial 3-day exposure to bacterial toxins, the cells were washed and replaced with conventional culture medium. Based on the previous results, LTA had no effect and therefore 1 μg/mL of LPS was chosen. Total cellular RNA was extracted from cultured cells using TRIzol (Invitrogen) according to the manufacturer's instructions. Reverse transcription was performed on the DNase I-treated RNA using random hexamers and RNase H- Superscript II reverse transcriptase (Invitrogen) according to the manufacturer's recommendation. The cDNA that was synthesized was subjected to PCR amplification, and the PCR product was quantified by real-time PCR using the Taqman fluorescence kit (Applied Biosystems, Foster City, CA). The primers that were used are shown in Table 2. The β-actin gene was amplified as an internal control. Standard curves were generated using serial dilutions of plasmids (~10–10^7 ^copies) containing the cloned sequences involved. To standardize the results for variability in RNA and cDNA quantity and quality, the results were converted to the number of target copies per 10^5 ^copies of β-actin gene.

**Table 2:** DNA sequences of primers used in quantitative PCR analysis

Gene	Sequence
β-actin	(F) GGA TGC AGA AGG AGA TCA CTG
	(R) CGA TCC ACA CGG AGT ACT TG
	(P) CCC TGG CAC CCA GCA CAA TG
TLR2	(F) TGT GAA GAG TGA GTG GTG CAA GT
	(R) ATG GCA GCA TCA TTG TTC TCA T
	(P) TGA ACT GGA CTT CTC CCA TTT CCG TCT TT
TLR4	(F) CAC TCG ATG TCA TTC CAA AGT TAT TG
	(R) AGA GTG CCC CCT TTA AAC AAA TT
	(P) TAC TAA GTA ATG ACT GTC ATG AAA GCA GCA T

F, forward primer; R, reverse primer; P, Taqman probe.

TLR2, Toll-like receptor 2; TLR4, Toll-like receptor 4

### Statistical analysis

All data are expressed as mean ± standard error of the mean. Differences between groups were analyzed by paired Student t tests or one-way ANOVA. A *P *value of < 0.05 was considered to be significant.

## Authors' contributions

GCFC originated the project, supervised the overall conduct of the research in his laboratory. IFYM carried out all the experimental work in this study, analyzed the data and draft the manuscript. HKWL helped in carrying out the TLR QPCR experiments, gave advice in data analysis and amended the manuscript. KHKY provided advice in the experimental design and contributed with part of the funding support. WKC helped in preparing the manuscript. YLL read and gave advice in the manuscript. All authors read and approved the final manuscript.
